# Neurofunctional correlates of eye to hand motor transfer

**DOI:** 10.1002/hbm.24969

**Published:** 2020-03-13

**Authors:** Cristián Modroño, Rosario Socas, Estefanía Hernández‐Martín, Julio Plata‐Bello, Francisco Marcano, José M. Pérez‐González, José L. González‐Mora

**Affiliations:** ^1^ Departamento de Ciencias Médicas Básicas (Unidad Departamental de Fisiología) Facultad de Ciencias de La Salud, Universidad de La Laguna (ULL), Campus de Ofra San Cristóbal de La Laguna (S/C de Tenerife) Spain; ^2^ Servicio de Rehabilitación, Hospital Universitario de Canarias San Cristóbal de La Laguna (S/C de Tenerife) Spain; ^3^ Servicio de Neurocirugía, Hospital Universitario de Canarias San Cristóbal de La Laguna (S/C de Tenerife) Spain; ^4^ Instituto Médico Tinerfeño San Cristóbal de La Laguna (S/C de Tenerife) Spain; ^5^ Instituto Universitario de Neurociencia San Cristóbal de La Laguna (S/C de Tenerife) Spain

**Keywords:** basal ganglia, brain mapping, cerebellum, eye movements, fMRI, motor activity, motor cortex, neurorehabilitation, transfer of learning, upper limb

## Abstract

This work investigates the transfer of motor learning from the eye to the hand and its neural correlates by using functional magnetic resonance imaging (fMRI) and a sensorimotor task consisting of the continuous tracking of a virtual target. In pretraining evaluation, all the participants (experimental and control group) performed the tracking task inside an MRI scanner using their right hand and a joystick. After which, the experimental group practiced an eye‐controlled version of the task for 5 days using an eye tracking system outside the MRI environment. Post‐training evaluation was done 1 week after the first scanning session, where all the participants were scanned again while repeating the manual pretraining task. Behavioral results show that the training in the eye‐controlled task produced a better performance not only in the eye‐controlled modality (motor learning) but also in the hand‐controlled modality (motor transfer). Neural results indicate that eye to hand motor transfer is supported by the motor cortex, the basal ganglia and the cerebellum, which is consistent with previous research focused on other effectors. These results may be of interest in neurorehabilitation to activate the motor systems and help in the recovery of motor functions in stroke or movement disorder patients.

## Graphical abstract

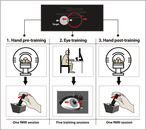

## 1 INTRODUCTION

The term motor skill learning refers to the increase in the accuracy of movements with practice (Willingham, 1998). This process is highly dependent on neural plasticity and can be divided into an early stage with fast improvements that occur within a single training session, and a late stage with slower improvements that occur over the course of multiple training sessions (Dayan & Cohen, 2011). Qualitative (Doyon et al., 2009; Doyon & Benali, 2005; Doyon, Penhune, & Ungerleider, 2003; Hikosaka, Nakamura, Sakai, & Nakahara, 2002) and quantitative (Hardwick, Rottschy, Miall, & Eickhoff, 2013) reviews of the neuroimaging literature confirm that motor skill learning is supported by cortical and noncortical motor areas such as the motor cortex, the basal ganglia and the cerebellum, whose interactions are decisive in facilitating the high cognitive and control requirements of the learning processes. It is interesting to note that motor learning has been related with both increasing and decreasing neural activations, where increasing activations may reflect the recruitment of additional neural substrates and decreasing activations suggest that the task can be carried out using fewer neuronal resources (Lustig, Shah, Seidler, & Reuter‐Lorenz, 2009; Poldrack, 2000).

A major outcome of motor learning is motor transfer, a concept which refers to the application of a learned skill in a new context (Censor, 2013). This ability to generalize skills to new conditions and task variants can be highly useful in daily life to optimize time and effort during the learning process, and has caught the interest of motor learning researchers for many years, giving rise to numerous behavioral studies (Adams, 1987).

One important kind of motor transfer happens when a learned task is performed with a new effector, different to that used in the training period. Previous research has reported motor skill transfer between effectors like hands, lower limbs, fingers, ankles, elbows, or eyes (Albano & Marrero, 1995; Christiansen, Larsen, Grey, Nielsen, & Lundbye‐Jensen, 2017; Krakauer, Mazzoni, Ghazizadeh, Ravindran, & Shadmehr, 2006; Plow & Carey, 2012; Schulze, Luders, & Jancke, 2002; Stoeckel & Wang, 2011). Intereffector transfer has been mainly studied when it happens between two corresponding contralateral limbs, which is also known as cross‐education (Ruddy & Carson, 2013), but it can also happen between different anatomical structures. For example, the hand can transfer motor skills to other organs, as has been observed for centuries in foot and mouth painters, who as a result of illness or accident have no use of their upper limbs. Nowadays, and especially in virtual environments, the eyes can be used to control elements with the help of eye tracking systems, which makes the eye a similar effector to the hand, thus different motor skills can probably be transferred from one to the other. To our knowledge, this kind of transfer has still not been studied and may be an interesting object of research whose results could be compared with those obtained at the interlimb level (basically, in cross‐education studies) to obtain information at the higher intereffector level. The study of eye to hand motor transfer also has potential implications for interactive technologies based on eye tracking techniques that are often applied in clinical and nonclinical environments; for example, to allow patients to control elements or to add an extra input method in videogames (Cognolato, Atzori, & Mueller, 2018; Levac, Huber, & Sternad, 2019).

On the other hand, despite the interest that motor transfer has aroused in behavioral research, there are few works on its neural correlates, which are still not well understood as several researchers have noted (Ruddy, Leemans, Woolley, Wenderoth, & Carson, 2017; Seidler & Noll, 2008; Uggetti et al., 2016). Therefore, it would be interesting to perform new studies to shed more light on this issue and allow further meta‐analysis. In this sense, one important observation that was derived from available neural studies some years ago (Anguera, Russell, Noll, & Seidler, 2007; Seidler, 2010; Seidler & Noll, 2008) is that motor transfer is associated with brain activations that are also characteristic of motor learning (especially in its late stage); an evaluation of this observation at the intereffector level could be helpful for the development or improvement of neural models of motor transfer. Furthermore, beyond the basic perspective, neural studies on motor transfer are also of interest in the field of neurorehabilitation because they can help to understand and establish new ways of generating activation in motor systems and support the recovery of motor functions of patients (Johansen‐Berg et al., 2002; Szameitat, Shen, Conforto, & Sterr, 2012), for example by using the eye instead of the hand to control virtual objects (Modroño et al., 2015).

**Figure 1:**
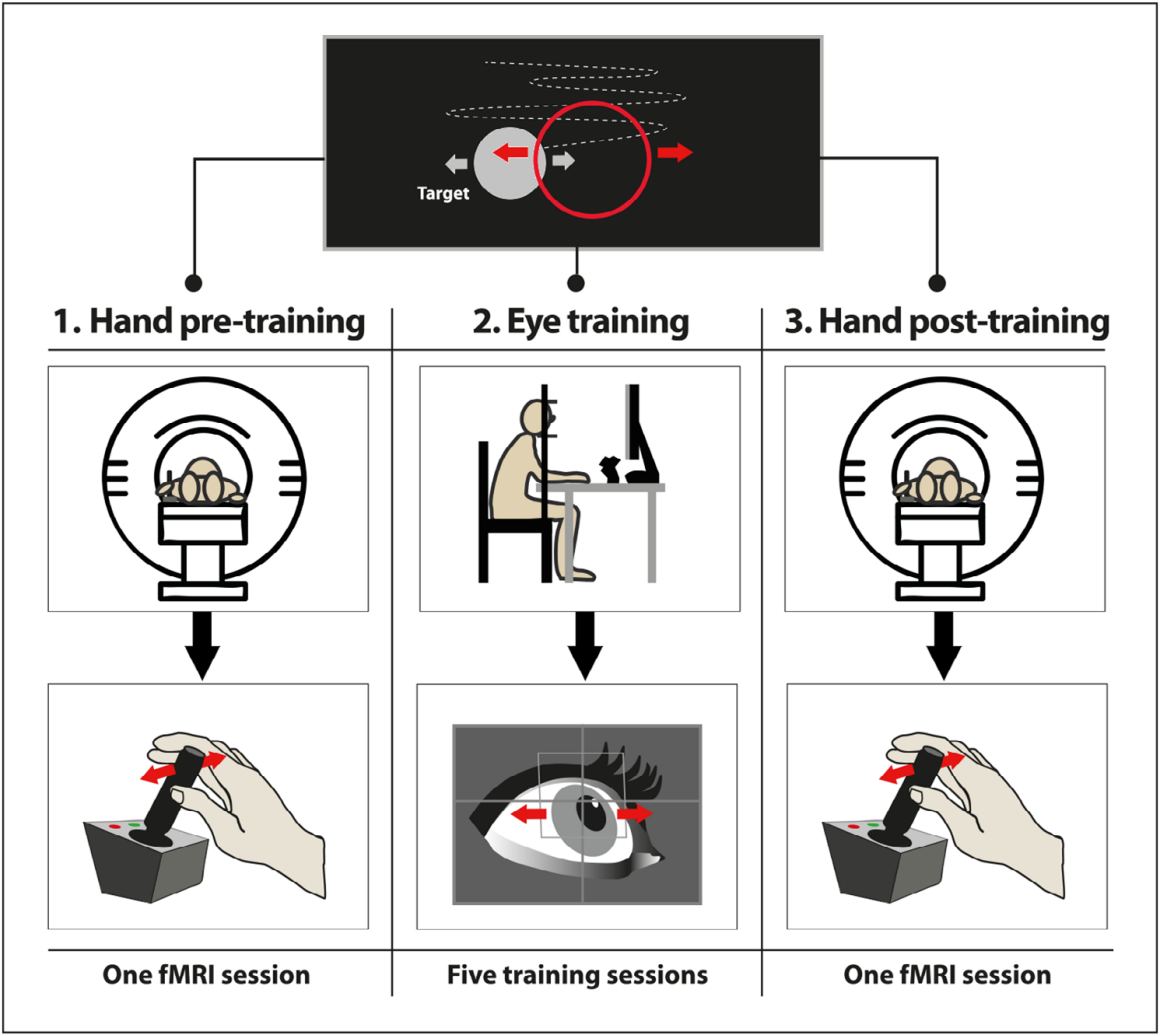
Experimental design. Participants controlled the red circle to track the gray circle (target) that was moving horizontally in a sine–cosine waveform (top of the figure). Gray/red arrows and dotted curve depict directions of movements and target trajectory, respectively (not visible during the task). In the pretraining and post‐training evaluation, participants performed the continuous tracking task using the right hand during fMRI acquisition. The experimental group also practiced an eye‐controlled version of the tracking task for 5 days outside the MRI scanner using an eye tracking system. fMRI, functional magnetic resonance imaging

Taking into account the interest of basic and applied neuroscience in understanding the neural mechanisms underlying motor transfer, and given the lack of specific works on eye to hand motor transfer, which has a potential application in neurorehabilitation, the aim of the present work is to study eye to hand motor transfer and its neural correlates by means of functional magnetic resonance imaging (fMRI) and a sensorimotor task where a virtual object is ocularly or manually controlled by healthy volunteers to pursue a moving target (Figure 1). On the basis of previous literature that has consistently shown the existence of motor transfer between other effectors (see above), we hypothesize that: (a) the training in the control of a virtual object with the eyes should lead to an increase in the accuracy of movements that (b) can be transferred to the hand. Furthermore, (c) one would expect the behavioral improvements transferred to the hand to be associated with neural activations in the motor system (because a large majority of papers in the motor literature report increases of activity with practice, we would expect to find increases of activation; however, a considerable percentage of studies report decreases of activation [Hardwick et al., 2013], thus we do not rule out finding this).

More specifically, and given that motor transfer is associated with brain activations that are also characteristic of motor learning (Seidler, 2010), activations can be expected in the cortical and subcortical regions that are most related to the learning of sensorimotor tasks. These regions are: the left dorsal premotor cortex (dPMC), the supplementary motor area (SMA), the primary motor cortex (M1), the putamen, and the cerebellum (Hardwick et al., 2013). Thus, at the cortical level, we predict activations in the left dPMC and in the SMA (during the learning period, the sensorimotor task is controlled with the eye and presumably the M1 is not involved [Modroño et al., 2015], thus, the M1 will not initially be proposed as a candidate to be activated at transfer). At the subcortical level, we predict activations in the putamen and the cerebellum. In any case, and because there is a tight coupling between experimental tasks and neuroimaging results, this set of candidates is not exclusive and other regions, especially in the sensorimotor system, may appear as being related to eye to hand motor transfer.

## 2 MATERIALS AND METHODS

### 2.1 Participants

Two groups of participants (experimental and control), recruited from university students, took part in the experiment. The experimental group consisted of 16 participants (13 female, 3 male) between 18 and 31 years of age (mean = 21.27; SD = 3.56). The control group was matched in gender and age to the experimental group: 16 participants (13 female, 3 male) between 18 and 30 years of age (mean = 21.77; SD = 2.84). No significant differences in age were found between groups (*t*(30) = −.44, *p* = .660). All the participants were right‐handed and neurologically healthy. They had normal or corrected‐to‐normal vision. They gave their written informed consent. The study was approved by the local Ethics Committee (University of La Laguna; registry number: CEIBA2015‐0178) and was conducted in accordance with the Declaration of Helsinki.

### 2.2 Task

Tracking tasks consist of a continuous input signal (the target) which a subject must try to match as closely as possible by his/her output response by controlling the position of a sensor. This kind of task is considered as a powerful tool to study the human sensory‐motor system (see Jones, 2015 for an interesting review), and has been used in different neuroimaging experiments (Limanowski, Kirilina, & Blankenburg, 2017). Behaviorally, an increase of accuracy in the tracking task would reflect improvements in generalized motor control (Meehan, Randhawa, Wessel, & Boyd, 2011).

In the present work, participants were engaged in a continuous tracking of a target moving horizontally in a sine–cosine waveform (Figure 1). The target appeared on a black screen as a gray circle and the participant had to track it with a red circle (cursor). The trajectories of these two virtual objects did not leave a trail. Custom software using Visual C# and DirectX was developed to implement the task. The sine–cosine pattern was constructed using the equation below with the following general form (Wulf & Schmidt, 1997), where coefficients were selected at random ranging from 5.0 to −5.0:

$$f\left(x\right)=b_{0}+a_{1}\;\sin\left(x\right)+b_{1}\;\cos\left(x\right)+a_{2}\;\sin\left(2x\right)+b_{2}\;\cos\left(2x\right)+\cdots +a_{6}\;\sin\left(6x\right)+b_{6}\;\cos\left(6x\right).$$

On the first day of scanning, all the participants performed the continuous tracking task inside the MRI scanner (pretraining evaluation) using their right hand and a joystick. After which, (only) the experimental group practiced the eye‐controlled version of the task for five consecutive working days outside the MRI scanner using an eye tracking system. One week after the first scanning session, all the participants were scanned again (post‐training evaluation), repeating the manual pretraining evaluation task. Previous works on neurophysiological changes associated to different modalities of motor practice have used similar training periods between two fMRI sessions (Lacourse, Orr, Cramer, & Cohen, 2005; Meehan et al., 2011; Parsons, Harrington, & Rao, 2005; Wadden, Brown, Maletsky, & Boyd, 2013). Details about scanning and training tasks are given below.

### 2.3 fMRI scanning

Participants were instructed to track the moving target as accurately as possible and to focus their gaze on the gray cross during the fixation blocks, without moving their hand. Participants were asked to move their head and trunk as little as possible during the experiment. After which they had a 5‐min practice session inside the MRI scanner to get familiar with the motor task. Participants performed the task by using an MRI‐compatible joystick (Resonance Technology, Inc., Northridge, CA). The fMRI run consisted of 10 tracking blocks of 20 s, separated by 20 s fixation blocks. The first and last blocks of the run were fixation blocks. The fMRI‐tracking task was the same for the pretraining evaluation and the post‐training evaluation, and all the participants used the same sets of (randomly generated) coefficients. Ten different sets of coefficients were generated for the fMRI‐tracking task (one per tracking block). Visual stimuli were given via MRI‐compatible eyeglasses (Visuastim, Resonance Technology, Inc.). The eyeglasses had a resolution of 800 × 600 pixels, 32 bit color depth and a refreshing rate of 60 Hz. The angle of vision corresponded to 30 × 22.5°. No audio stimuli were delivered. Logs were recorded for further analysis. The logs from one participant of the experimental group were lost due to technical problems. As a supplementary behavioral measure, eye movements were recorded by using an MRI‐compatible eye tracking system (MReyetracking, Resonance Technology Company, Northridge, CA), which tracked the participant's gaze point with a temporal resolution of 30 Hz.

### 2.4 Motor training

After the first MRI scanning, participants belonging to the experimental group were scheduled for five training sessions in a behavioral, quiet laboratory. During the practice days, participants were seated in front of a computer monitor (U2410 24″, Dell Technologies, Round Rock, TX) and used their gaze to perform the tracking task. This was done by using an eye tracking system (Eye Tribe Tracker, The Eye Tribe ApS, Copenhagen, Denmark) which tracks the participant's gaze point (i.e., where they are looking) in real‐time (sampling rate: 30 Hz; accuracy: 0.5° (average); spatial resolution: 0.1° [RMS]; operating range: 45–75 cm). This system includes a Software Developer's Kit (SDK) that allowed our custom developed task to seamlessly interface with the eye tracker. Using this SDK, the raw gaze point horizontal coordinates were linearly transformed into positions of the tracking circle, which allowed the participant to control it in real‐time. Logs were recorded for further analysis.

The eye tracking system was calibrated following the instructions of the user manual for each participant and training session, and head position was fixed using a forehead support, at a distance of 65 cm from the screen. The light and temperature of the laboratory and participant's position were similar throughout the experiment. All participants performed the training in the same manner.

Participants performed six training runs per day (with a brief resting period between runs where they were allowed to move to favor their comfortability). Each training run consisted of 10 tracking blocks of 30 s; the tracking blocks where separated by a 7.5 s fixation screen. This makes a total of 300 training blocks per participant (5 days/participant × 6 runs/day × 10 blocks/run), and the overall training time was 2.5 hr. During the training period, a different set of coefficients was randomly generated for each block for each run, day, and participant.

### 2.5 MR image acquisition

Axially oriented functional images were obtained by a 3T Signa HD MR scanner (GE Healthcare, Waukesha, WI) using an echo‐planar‐imaging gradient‐echo sequence and an eight channel head coil (TR = 2000 ms, TE = 22 ms, flip angle = 75°, matrix size = 64 × 64 pixels, 36 slices, 4 × 4 mm in plane resolution, spacing between slices = 4 mm, slice thickness = 3.3 mm, interleaved acquisition). The slices were aligned to the anterior commissure‐posterior commissure line and covered the whole brain. Functional scanning was preceded by 18 s of dummy scans to ensure tissue steady‐state magnetization. A total of 210 volumes were taken during each of the two runs (pretraining evaluation/post‐training evaluation) for every participant. High resolution sagittally oriented anatomical images were also collected for anatomical reference. A 3D fast spoiled‐gradient‐recalled pulse sequence was obtained (TR = 8.8 ms, TE = 1.7 ms, flip angle = 10°, matrix size = 256 × 256 pixels, 1 × 1 mm in plane resolution, spacing between slices = 1 mm plus 0 mm interslice gap, slice thickness = 1 mm).

### 2.6 Image preprocessing and analysis

Images were checked for artifacts and then analyzed using SPM12 software (www.fil.ion.ucl.ac.uk/spm/). The functional images were realigned to their mean image and then unwarped to remove residual head motion related variance (Andersson, Hutton, Ashburner, Turner, & Friston, 2001), and normalized to the MNI space. Normalization success was validated by visual inspection. The normalized images of 2 × 2 × 2 mm were smoothed by a full width at half maximum (FWHM) 8 × 8 × 8 Gaussian kernel.

A block design in the context of a general linear model was used, for individual subject analyses (first level), to look for differences in brain activity during the tracking periods and the fixation periods. To model the BOLD response in each experimental condition, the first level design matrix included two sessions (pretraining evaluation and post‐training evaluation) with two conditions each (tracking and fixation). Thus, four conditions were included in the design matrix: *tracking‐pre*, *fixation‐pre*, *tracking‐post*, and *fixation‐post*. The conditions were modeled using a box‐car function convolved with the hemodynamic response function (HRF). A temporal high‐pass filter (128 s) was applied to remove slow signal drifts. Activation maps were generated for each subject by applying t statistics. Two contrasts of interest were computed at the first level: *tracking‐pre > fixation‐pre* and *tracking‐post > fixation‐post*.

The first‐level contrast images were then used in a random‐effects group analysis (second level) to model a two‐way repeated measures ANOVA. Analysis was performed using a two (group: *ocular train*, *control*) × 2 (day: *hand pretraining evaluation*, *hand post‐training evaluation*) full factorial design (i.e., in SPM, Full Factorial Design; using group and day as between‐subject and within‐subject factors, respectively). Directional contrasts (SPM *t* contrasts) were then applied to the ANOVA parameter estimates to test (a) the interaction between group and day and (b) the simple effects of day in each group.

### 2.7 Behavioral outcome measures and analysis

Motor performance was evaluated using the *mean absolute error* (MAE). This measure indicates the mean average distance the tracking circle was away from the target irrespective of the side, and is the most commonly used measure of overall accuracy in tracking tasks (Jones, 2015). Departing from the eye and the manual logs, tracking *mean absolute error* was calculated for the pretraining evaluation and the post‐training evaluation of each participant, and also for every practice day in the subjects of the experimental group. Changes in accuracy related to the practice sessions were analyzed using analysis of variance in two different ways: intragroup for the eye‐controlled task (experimental group) and between groups for the hand‐controlled task (see below).

## 3 RESULTS

### 3.1 Behavioral

**Figure 2:**
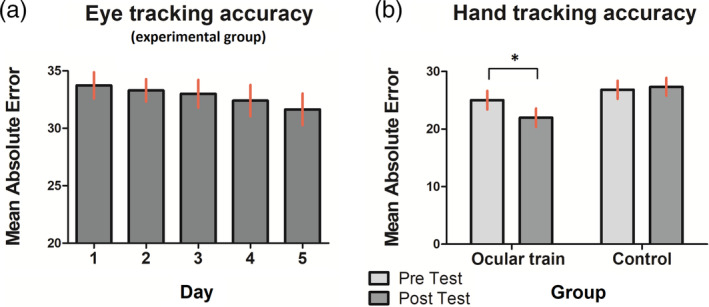
(a) Eye tracking accuracy scores show a better performance across training sessions for the ocular train group (*p* < .05). (b) Hand tracking accuracy scores show a better performance for the ocular train group during the post‐training evaluation. This performance improvement was not observed in the control group. *p < .001. Error bars depict *SE*

In the first place, to test whether the practice of the eye‐controlled tracking task was related with a better performance in the same modality of control, we performed a one‐way ANOVA with five repeated measures using the ocular accuracy scores of the experimental group. Figure 2a shows a continuous *mean absolute error* decrease (i.e., a better performance) across training sessions. This was confirmed by linear within‐subjects contrast: (*F*_1,15_ = 6.45, *p* = .023, *η*_p_^2^ = .301).

In second place, we tested for possible differences in the manual accuracy score using a two (group: *ocular train*, *control*) × 2 (day: *hand pretraining evaluation*, *hand post‐training evaluation*) repeated measures ANOVA (performance measures can be seen in Figure 2b). An interaction effect was found between group and day (*F*_1,29_ = 12.24, *p* = .002, *η*_p_^2^ = .297). Subsequent analyses showed a simple effect of day for the *ocular train* group (*F*_1,14_ = 20.27, *p* < .001, *η*_p_^2^ = .592) but not for the *control* group (*F*_1,15_ = .469, *p* = .504, *η*_p_^2^ = .030). The decrease of the *mean absolute error* in the trained group indicates that the practice of the eye‐controlled task resulted in a better performance in the hand‐controlled task; this tracking improvement did not happen in the untrained (control) group.

Visual inspections of the MRI‐compatible eye tracking records showed that during the manual task, participants were also following the target with their gaze, which is common behavior in this kind of visuomotor tasks (Danion & Flanagan, 2018).

### 3.2 Neural

#### 3.2.1 Activations during the hand tracking task

**Figure 3:**
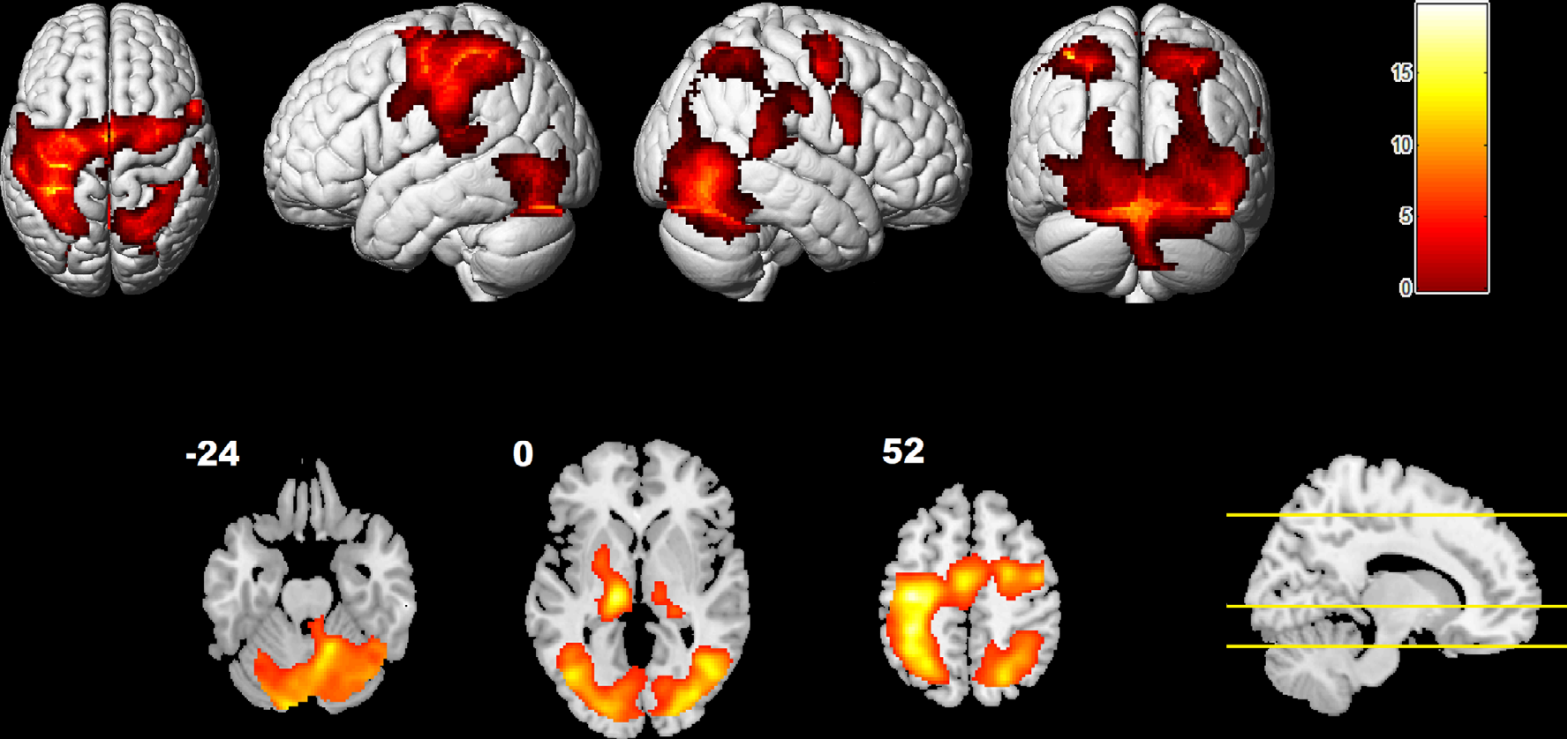
Brain rendering and three axial slice sections (selected at the height of motor cortical, subcortical, and cerebellar regions) showing activations for the all tracking conditions > fixation contrast across all participants (*p* < .05, FWE voxel‐wise corrected). Numbers near the slices depict the Z MNI coordinate

In order to characterize brain regions activated during the hand tracking task across all participants and factors independently of the experimental manipulation, all the tracking conditions were compared with the fixation baseline (statistical maps were set at a voxel‐level threshold of *p* < .05, family‐wise error rate (FWE) corrected, *k* = 25 voxels). A statistical parametric map resulting from this contrast was displayed on the surface and on three selected axial slices of a MNI single‐subject T1 image (Figure 3). The results revealed activations in cortical and noncortical regions typically involved in visuomotor tasks (Modroño et al., 2015; Modroño, Navarrete, Rodriguez‐Hernandez, & Gonzalez‐Mora, 2013), including the supplementary motor area, the premotor cortex, the primary motor cortex, the basal ganglia, the thalamus, parieto‐occipital regions and the cerebellum. Activations were found in the left (but not in the right) primary motor cortex, which is consistent with the fact that the task was performed with the right hand.

#### 3.2.2 Effects of the ocular training (group × day interaction)

**Figure 4:**
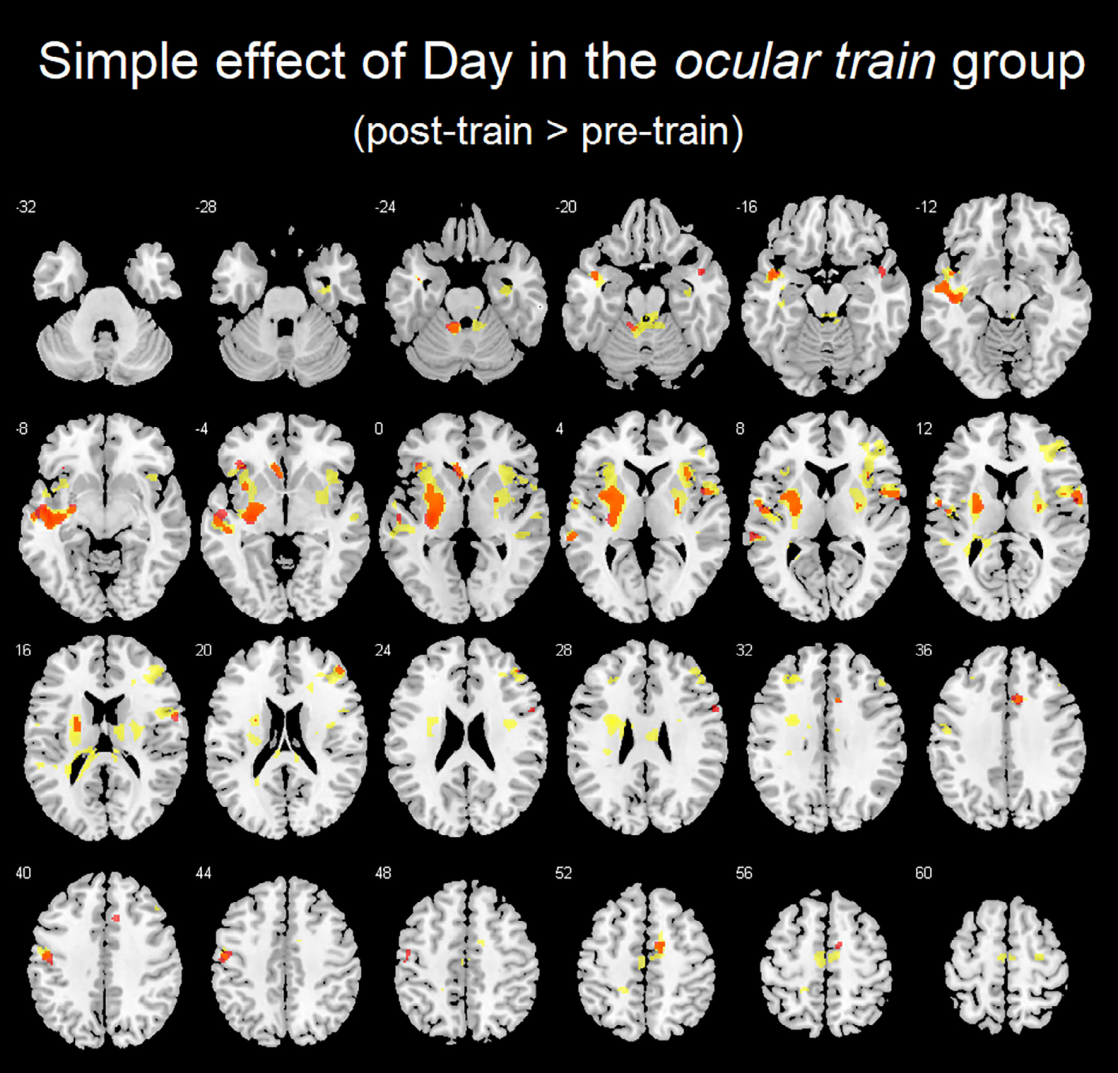
Neural activations associated to the transfer of motor learning from the eye to the hand. Red voxels: significant activation clusters resulting from the simple effect of day in the ocular train group (post‐training evaluation > pretraining evaluation; no activations were found for the opposite contrast). Yellow voxels: significant activation clusters resulting from the group × day interaction. Common voxels of both contrasts are shown in orange (virtually the same voxels obtained for the simple effect of day); many of which are located in motor regions (e.g., precentral gyrus, basal ganglia, and cerebellum). No significant simple effects of day were found for the control group. Threshold: *p* < .05 FDR corrected at the voxel level, *k* = 25 (simple effect of day was inclusively masked with the group × day interaction contrast; uncorrected mask *p*‐value = .05)

An important goal of the present study was to investigate the changes of neural activity associated with the training in the eye‐controlled task. With this aim in mind, we tested the interaction between group and day (statistical maps were set at a voxel‐level threshold of *p* < .05, false discovery rate (FDR) corrected, *k* = 25 voxels). Interestingly, we found significant interaction effects in a number of clusters, many of them located in motor regions, such as the cerebellum, the basal ganglia, the precentral gyrus and the insular cortex (Figure 4). The post hoc comparisons that followed from the interaction resulted in a significant simple effect of day in the *ocular train* group (*p* < .05, FDR corrected at the voxel level, *k* = 25; inclusively masked with the interaction contrast, uncorrected mask *p*‐value = .05), showing increases of activity during the post‐training evaluation in the abovementioned motor regions (Table 1, Figure 4). No significant decreases of activity were found. As regards the *control* group, no significant simple effects of day were found.

**Table 1:** Main anatomical structures activated for the simple effect of day in the *ocular train* group (post‐training evaluation > pretraining evaluation)

Region	BA	Cluster size (voxels)	[*X Y Z*]	Peak *T* value
Left hemisphere				
Inferior frontal gyrus	45	55	−38 30 –4	3.85
Precentral gyrus	6	80	−52 –10 8	4.03
Precentral gyrus	6	86	−50 –10 42	4.19
Superior temporal gyrus	38	68	−46 2 –16	4.44
Superior temporal gyrus	22	47	−62 –30 4	3.92
Putamen, globus pallidus, insula, middle/superior temporal gyrus	21, 22	1,094	−48 −14 −10	6.13
Caudate, anterior cingulate	24, 25	57	−8 24 –2	4.31
Cerebellum anterior lobe		69	−10 −44 −24	4.04
Right hemisphere				
Inferior frontal gyrus	45	27	56 10 26	3.99
Middle frontal gyrus	46	25	44 44 20	3.84
Middle cingulate	32	57	10 18 36	4.35
Precentral gyrus	6	145	58 2 12	4.00
Supplementary motor area	6	44	10 –2 54	3.94
Superior temporal gyrus	38	45	46 4 –20	4.50
Putamen, globus pallidus		44	26 –8 12	3.50
Insula	13	34	34 20 6	3.56

*Note:* Anatomical structures and Brodmann areas (BA) are shown with corresponding MNI coordinates of peak activity in each cluster. Threshold: *p* < .05 FDR corrected at the voxel level, *k* = 25 (the simple effect of day has been inclusively masked with the group × day interaction contrast; uncorrected mask *p*‐value = .05).

It has been noticed that, in addition to reflecting learning related changes, differences in activation between pretraining and post‐training may reflect the differences in performance that occur with practice (Poldrack, 2000; Seidler, 2010). To control such performance effects on brain activations, two additional SPM models were created and tested. These models were similar to the previous one but included one of two different performance covariates in the full factorial design. The covariates were the *mean absolute error* (see above) and the total cursor displacement (calculated as the addition of the Euclidean distances between the cursor position of each sample and the cursor position of the following sample). The analyses performed for the previous model were repeated for the new ones, showing interactions and simple effects that were very similar to those obtained for the previous one (Figure S1, Supporting Information). Furthermore, the results did not show any positive or negative relationship between any of the two performance covariates and brain activity (*p* < .05, FDR corrected at the voxel level, *k* = 5).

## 4 DISCUSSION

From a neurobehavioral perspective, besides being sensory organs, the eyes can be considered as effectors because they are able to move in response to a stimulus. However, on their own, eyes do not have the capability to make relevant changes in the environment as other effectors (e.g., the hands) do. This capability can be dramatically enhanced with the help of an eye tracking system, especially in digital environments. In such an environment, the eyes can be used to control virtual objects in a similar way as a hand does when using a joystick or a computer mouse. Thus, qualitatively speaking, the eye and hand can be similar effectors within a virtual setting, and acquired motor skills can probably be transferred from one to the other.

The above issue has been addressed in the present work by using a sensorimotor tracking task where a virtual object was ocularly or manually controlled by the participants. On the basis of previous literature, it was hypothesized that: (a) the training in the control of a virtual object with the eyes should lead to an increase in the accuracy of movements that (b) can be transferred to the hand. Furthermore, the behavioral improvements transferred to the hand were expected to be associated with neural activations in the motor system (c), specifically, in the motor cortex, the putamen and the cerebellum.

At the behavioral level, it was found that the practice in the eye‐controlled version of the tracking task was related with a better performance in such a modality of motor control (Figure 2a). This result is consistent with those obtained in many related studies where the participants were trained in similar sensorimotor tracking tasks but using the hand as an effector (Ewolds, Broeker, de Oliveira, Raab, & Kuenzell, 2017; Lang, Gapenne, Aubert, & Ferrel‐Chapus, 2013; Meehan et al., 2011; 2013; Wadden et al., 2013; 2015; 2017; Zhu et al., 2014). These tracking tasks require different perceptive, executive and motor functions (e.g., visuospatial analysis, visuospatial attention, visuomotor association, motor execution, or motor programming) (Lutz, Martin, & Jaencke, 2010), and performance improvements occur as participants learn new kinematics or dynamics. The acquisition of motor skills has also been associated with the formation and modification of internal models of movement, which could then be used to anticipate task‐specific requirements (Shadmehr & Holcomb, 1997). In the experiment here, the effector was the eye instead of the hand during the training period, but similar processes must have taken place to enable the ocular learning shown by the results.

Consistent with previous research which has found motor skill transfer between different effectors (Christiansen et al., 2017; Krakauer et al., 2006; Plow & Carey, 2012; Schulze et al., 2002; Stoeckel & Wang, 2011), the results presented here show that practice in the eye‐controlled version of the tracking task was related with a better performance in the hand‐controlled modality of such a task; in other words, there was a motor transfer between the eye and the hand. Motor transfer has been associated with diverse and complex processes (Seidler, 2010), which in the present case may include, among others, retrieval and modifications of the internal models that were acquired during the eye training period, and the formation of new ones. Such processes would be reflected in the neural activations accompanying the improvements in hand tracking performance and that have been found in most of the motor regions proposed in our hypothesis (SMA, putamen, and cerebellum), as discussed below. It should be noted that these activations can reflect not only adaptive processes like those mentioned above, but also the differing performance levels that occur with practice (Poldrack, 2000; Seidler, 2010). However, the analyses performed with the additional SPM models do not indicate that this is the case, since the results are similar after regressing out the performance measures.

Activations at transfer were observed in the SMA. This region is involved in different aspects of motor learning (Nachev, Kennard, & Husain, 2008; Ruan et al., 2018), including intertask (Auer, Dewiputri, Frahm, & Schweizer, 2018; Parsons et al., 2005) and, which is more related with the present experiment, interlimb motor transfer (Jung, Park, Kim, & You, 2019; Perez et al., 2007; Ruddy et al., 2017). In this regard, the SMA has been associated with functions relevant to such interlimb transfer, like interhemispheric motor control (e.g., influence in the control of an untrained moving limb during transfer) or the prevention of unwanted movements (of a limb that should be static during the transfer test). The present results can be considered as an extension of previous ones, showing that the SMA plays a role not only in interlimb but also, at a more general level, in intereffector transfer. Such a role seems to be mediated by the SMA position and its connectivity with several motor regions (thalamus, basal ganglia, cerebellum) usually activated in transfer experiments (Ruddy et al., 2017), including the one presented here (see below).

The most extended activations at transfer appeared in the basal ganglia, particularly in the (left) putamen, and to a lesser extent in the caudate and the globus pallidus, regions with a critical role in planning, executing, and learning a new motor skill (Doyon et al., 2009). As happens with the SMA, finding activations in the basal ganglia is consistent with the results of studies of interlimb motor transfer (Perez et al., 2007; Thut et al., 1997; Walz et al., 2015). In the present case, the basal ganglia activations may be related with several processes that are relevant to enhancing manual tracking performance, like the encoding of new motor programs for the hand (the striatum plays a critical role here) (Doyon et al., 2009), the automatization of hand movements (Walz et al., 2015), or online adjustments of movements that are necessary to track the target (Perez et al., 2007).

Functionally related with the basal ganglia during motor learning (Doyon et al., 2009), the cerebellum also showed increases of activity at transfer in the present experiment. Cerebellar activations are usually observed during the learning of sensorimotor tasks (Hardwick et al., 2013) and have been related with feed‐forward models for which sensory input is used prior to movement execution to improve movement accuracy (Wolpert, Miall, & Kawato, 1998). Such internal models are neural systems that mimic the behavior of the sensorimotor system and objects in the external environment; for example, in the present case, the ocular training phase may involve the creation of kinematic or dynamic models related to the movements of the target, the cursor, and the eye. During the transfer test, the old models might be used directly (e.g., those related with the movements of the target, because such movements are equivalent in the training and the transfer phases) or to implement new ones that are useful in the new context, for example, to link the control of the eye and the hand (Miall & Jenkinson, 2005), which may be reflected in cerebellar activations. This result is also consistent with previous transfer literature, where the cerebellum is one of the regions that is more frequently involved across studies (Jung et al., 2019; Lutz, Weidner, Shah, & Jancke, 2001; Matsumura et al., 2004; Obayashi, 2004; Parsons et al., 2005; Seidler & Noll, 2008; Shimizu, Wu, & Knowlton, 2016; Uggetti et al., 2016; Walz et al., 2015).

Because both the cerebellum and basal ganglia are connected to the cortical motor areas (and specifically to the SMA) in loops mediated by the thalamus (Jung et al., 2019), it is not surprising that during the transfer test activations have appeared in thalamic regions, which are also frequently involved in motor learning (Hardwick et al., 2013). Specifically, activity appeared in the anterior and posterior parts of the ventral lateral nucleus (VLa and VLp) and also in the ventral anterior nucleus (VA) (note that the VLp nucleus channels information from the cerebellum and the VLa and VA nuclei does this from the basal ganglia [Perez et al., 2007]). The present results are also consistent with studies performed at the interlimb level that relate cross transfer with a neural network formed by the SMA, the basal ganglia, the cerebellum and the thalamus (Jung et al., 2019; Perez et al., 2007), and this seems to indicate that at a higher intereffector level, motor transfer is also supported by these cortical and subcortical motor regions working in conjunction (Bostan & Strick, 2018). These results also support, at the intereffector level, the observation of Seidler (2010) regarding motor transfer being associated with brain activations that are also characteristic of motor learning, which could be useful to take into account to develop or improve neural models of motor transfer and also to guide further experiments.

We had also predicted activations at transfer in the left dPMC, due to the fact that it is a key node for motor learning (Hardwick et al., 2013). However, our analysis has not revealed activations there. This might simply be due to a lack of statistical power that may be overcome by using a larger sample size. Another option is to take a deeper look at the neural studies of motor transfer. Consistent with Hardwick's meta‐analysis on motor learning, such studies usually report left dPMC activations during the learning phase; however, at transfer, some of the studies also report these activations (Parsons et al., 2005; Shimizu et al., 2016), while others do not (Anguera et al., 2007; Grafton, Hazeltine, & Ivry, 2002; Thut et al., 1997). It should be noted that the former works have studied intertask transfer while the latter have studied intereffector transfer, and this may be a key difference for the lack of dPMC activations at transfer, in the latter works and in the present case. A possible explanation for this potential dissociation is that the intertask transfer paradigms might be more cognitively demanding than the intereffector paradigms and those cognitive demands may be supported by the dPMC, which can act as an interface between motor control and cognition (Hardwick et al., 2015).

Beyond the predicted activations, activations were also found in other cortical regions involved in motor functions, like the ventral premotor cortex, the cingulate cortex and the insular cortex (Anderson et al., 1994; Chouinard & Paus, 2006; Fink, Frackowiak, Pietrzyk, & Passingham, 1997; Kantak, Stinear, Buch, & Cohen, 2012; Loh, Hadj‐Bouziane, Petrides, Procyk, & Amiez, 2018). Despite the fact that these regions were not specifically mentioned in our hypothesis, this is not a surprising result since different studies have involved them in motor learning and motor transfer (Auer et al., 2018; Kantak et al., 2012; Lutz et al., 2001; Parsons et al., 2005; Ruddy et al., 2017; Wadden et al., 2013), and may support functions that are relevant to the experimental task here performed. It should also be mentioned that no decreases of activation were found in any brain region; however, this is not a surprising result bearing in mind the previous motor learning literature that often only reports increases of activation (Hardwick et al., 2013). The variability in the direction (positive or negative) of the activations in these kinds of studies is possibly related with temporal aspects of the experimental tasks, since the motor systems can show increases or decreases of activation that are dependent on the stage of motor learning (Nezafat, Shadmehr, & Holcomb, 2001).

In addition to the mentioned literature, some studies of smooth pursuit eye movements are helpful to understand the results presented here. In this regard, it is worth mentioning that when the arm and eyes execute together a tracking task, the performance of both systems is better than when they execute it separately (Danion & Flanagan, 2018; Koken & Erkelens, 1992; Miall & Reckess, 2002; Niehorster, Siu, & Li, 2015). This effect has been attributed to two different processes: (a) an interchange of signals between separate arm and eye control systems (Gauthier, Vercher, Ivaldi, & Marchetti, 1988; Lazzari, Vercher, & Buizza, 1997; Scarchilli & Vercher, 1999) (possibly supported by the cerebellum [Miall & Reckess, 2002]), and (b) a neural controller shared by both systems (Engel, Anderson, & Soechting, 2000; Maioli & Falciati, 2015; Maioli, Falciati, & Gianesini, 2007) (it has even been suggested that when moving the eyes alone, this common motor controller could be producing a motor plan for manual tracking [Maioli et al., 2007]). For the present case, both kinds of processes could be involved simultaneously: (a) during the post‐test (or even during the eye practice period), eye control systems couple to untrained hand control systems, exchanging information that promotes transfer, and (b) during the eye practice period, a common neural controller is being trained and this promotes the transfer observed later during the post‐test. In any case, other different processes could have affected the results presented here and more studies are needed to increase the limited knowledge on the neural bases of intereffector transfer.

Here it should be noted that during the post‐test, participants were tracking the target with their hand, but, as is usual in these kinds of tasks and as was shown by the MRI‐compatible eye tracking logs, they were also following the target with their eyes. Thus, it could be argued that the increases of activity found in the experimental group could simply be reflecting additional oculomotor practice in the task instead of motor transfer. However, the literature supports the idea that the activations are due to motor transfer: first, the previous neural studies on learning of smooth pursuit eye movements have not involved the basal ganglia (Burke & Barnes, 2008; Gonzalez, Billington, & Burke, 2016; Gonzalez & Burke, 2018; Kleiser, Stadler, Wimmer, Matyas, & Seitz, 2017; Maquet, Schwartz, Passingham, & Frith, 2003; Schmid, Rees, Frith, & Barnes, 2001), which is the region where the most prominent activations have appeared in the present case. Conversely, motor transfer studies have frequently involved the basal ganglia, as mentioned above. Thus, the extended activations found in the basal regions seem to be due to motor transfer. The left lateralization of the results, and the general consistence of the activated regions outside the basal ganglia with those described in previous transfer studies (see above) also points to the transfer of motor learning to the active right limb (Walz et al., 2015). Thus, although we do not preclude the possibility that the additional oculomotor practice could be driving the results to a certain extent, it seems reasonable to think that eye to hand motor transfer plays a fundamental role in the activations found. Finally, it should be noted that oculomotor practice could also drive the neural results of other kinds of transfer experiments (e.g., cross‐limb studies using a visuomotor tracking task), thus future works could include new experimental conditions (e.g., with the eyes of the participants fixated) to go into this point in greater depth.

The neural results presented here may also be of interest in clinical settings. In a previous fMRI work (Modroño et al., 2015) we studied brain activity associated to both the ocular and the manual control of a virtual object (note the above study was not a learning study as is the case here). The results obtained in the above study showed extended activations in sensorimotor areas that were similar regardless of what the effector was. Since activating the sensorimotor cortex benefits the recovery of functions in motor deficits (Johansen‐Berg et al., 2002; Szameitat et al., 2012), we suggested ocular control of virtual objects as a potential new approach to neurorehabilitation. The present study provides new evidence supporting this approach that could be applied in stroke patients whose motor areas are often affected, and perhaps even in movement disorders such as Parkinson's Disease where basal ganglia activity is usually affected (Herz, Eickhoff, Lokkegaard, & Siebner, 2014). To test this idea, and taking into account that eye tracking can be a nonexpensive technique, it would be worthwhile performing further experiments with clinical populations.

## 5 CONCLUSIONS

The present work shows that acquired ocular motor skills (specifically, the ocular control of virtual objects) can be transferred to the hand after a few sessions of eye training. This intereffector transfer has been accompanied by increases of neural activity in cortical and subcortical motor regions. In the context of the motor learning and motor transfer literature, particularly neuroimaging studies showing that similar regions contribute to interlimb motor transfer, the results presented here indicate that the motor cortex, the basal ganglia and the cerebellum support eye to hand motor transfer. These results may be of interest in neurorehabilitation to activate the motor systems and help in the recovery of motor functions in stroke or movement disorder patients.

## Supporting information


**Figure S1** The glass‐brain projections show significant activation clusters resulting from the group × day interaction and from the simple effect of day in the ocular train group (post‐training evaluation > pretraining evaluation). Results were obtained from three different SPM full factorial designs. Model one did not include performance covariates (it corresponds to the figures and table reported in the main manuscript). Model two included the mean absolute error as performance covariate. Model three included the total cursor displacement as performance covariate. Note that the results are similar for the three models. (*p* < .05, FDR corrected at the voxel level, *k* = 5; missing performance data from one participant was imputed by mean substitution [if this participant was eliminated from models two and three, similar results were obtained]).

## Data Availability

The data that support the findings of this study are available from the corresponding author upon request.
