# Supplementary material for: Neurofunctional correlates of eye to hand motor transfer
Source: Hum Brain Mapp. 2020 Mar 13;41(10):2656–68. doi: 10.1002/hbm.24969 (PMC7294058; doi:10.1002/hbm.24969)
Supplement: Supplementary file 1 — Figure S1 The glass‐brain projections show significant activation clusters resulting from the group × day interaction and from the simple effect of day in the ocular train group (post‐training evaluation > pretraining evaluation). Results were obtained from three different SPM full factorial designs. Model one did not include performance covariates (it corresponds to the figures and table reported in the main manuscript). Model two included the mean absolute error as performance covariate. Model three included the total cursor displacement as performance covariate. Note that the results are similar for the three models. (p < .05, FDR corrected at the voxel level, k = 5; missing performance data from one participant was imputed by mean substitution [if this participant was eliminated from models two and three, similar results were obtained]). [file HBM-41-2656-s001.docx]

Supplementary Materials

**
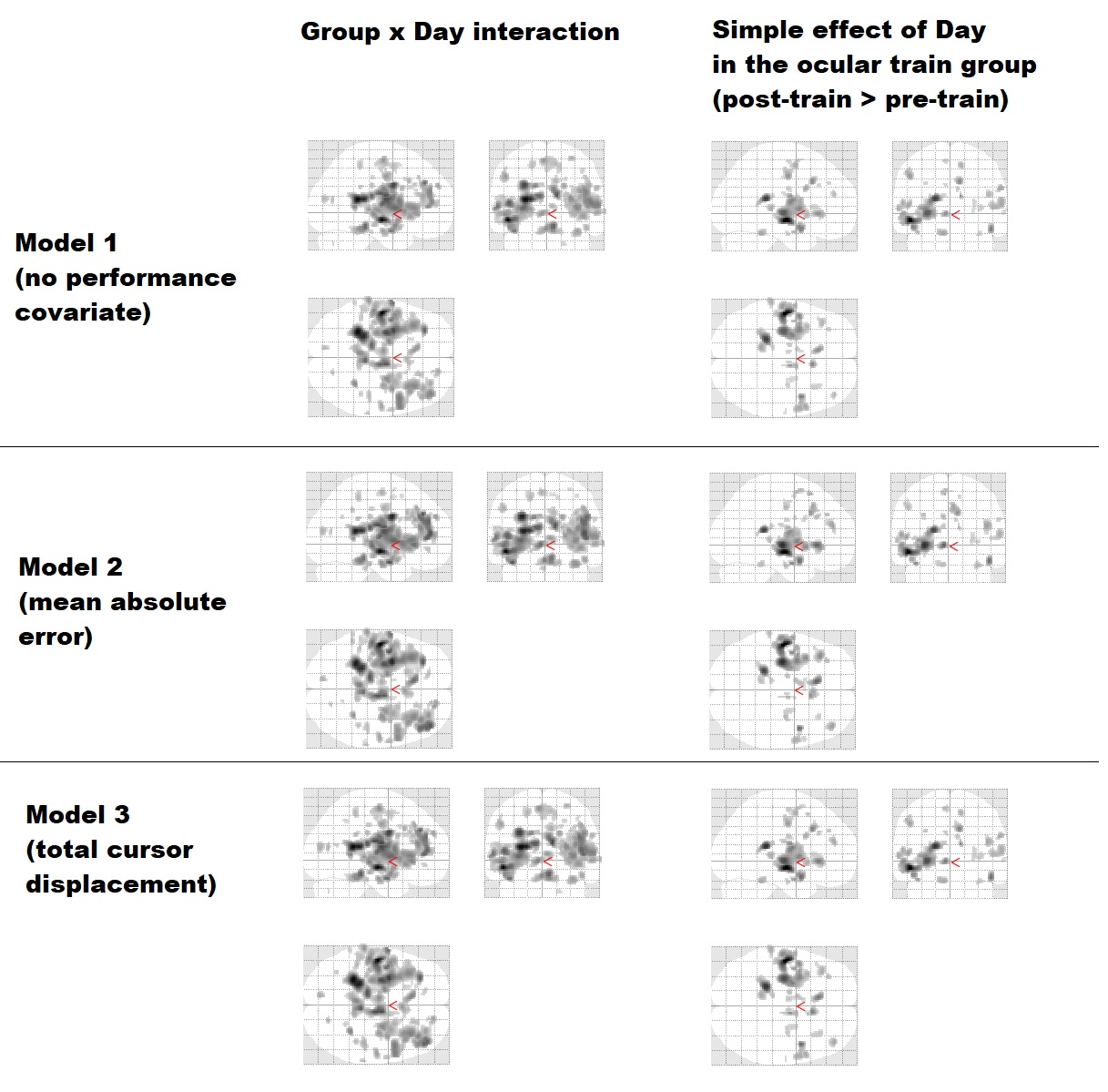
**

**Figure S1.** The glass-brain projections show significant activation clusters resulting from the Group x Day interaction and from the simple effect of Day in the ocular train group (post-training evaluation > pre-training evaluation). Results were obtained from three different SPM full factorial designs. Model one did not include performance covariates (it corresponds to the figures and table reported in the main manuscript). Model two included the mean absolute error as performance covariate. Model three included the total cursor displacement as performance covariate. Note that the results are similar for the three models. [p<0.05, FDR corrected at the voxel-level, k=5; missing performance data from one participant was imputed by mean substitution (if this participant was eliminated from models two and three, similar results were obtained)].
